# HDAC3 Knockdown Dysregulates Juvenile Hormone and Apoptosis-Related Genes in *Helicoverpa armigera*

**DOI:** 10.3390/ijms232314820

**Published:** 2022-11-26

**Authors:** Huimin Chang, Zhenlu Xu, Wenkang Li, Chenggu Cai, Wenjing Wang, Pengliang Ge, Xue Jia, Yingge Li, Tianze Ding, Wei Ma, Ali Mohammad Banaei-Moghaddam, Huijuan Mo, Maozhi Ren

**Affiliations:** 1 Zhengzhou Research Base, State Key Laboratory of Cotton Biology, School of Agricultural Sciences, Zhengzhou University, Zhengzhou 450000, China; 2 State Key Laboratory of Cotton Biology, Institute of Cotton Research, Chinese Academy of Agricultural Sciences, Anyang 455000, China; 3 Institute of Urban Agriculture, Chinese Academy of Agricultural Sciences, Chengdu 610000, China; 4 College of Plant Science and Technology, Huazhong Agricultural University, Wuhan 430000, China; 5 Science Center for Future Foods, Jiangnan University, Wuxi 214122, China; 6 Laboratory of Genomics and Epigenomics (LGE), Institute of Biochemistry and Biophysics (IBB), University of Tehran, Tehran 009821, Iran

**Keywords:** histone deacetylase 3, *H. armigera*, RNA interference, RGFP966, pupation, pest control

## Abstract

Insect development requires genes to be expressed in strict spatiotemporal order. The dynamic regulation of genes involved in insect development is partly orchestrated by the histone acetylation–deacetylation via histone acetyltransferases (HATs) and histone deacetylases (HDACs). Although histone deacetylase 3 (HDAC3) is required for mice during early embryonic development, its functions in *Helicoverpa armigera* (*H. armigera*) and its potential to be used as a target of insecticides remain unclear. We treated *H. armigera* with HDAC3 siRNA and RGFP966, a specific inhibitor, examining how the HDAC3 loss-of-function affects growth and development. HDAC3 siRNA and RGFP966 treatment increased mortality at each growth stage and altered metamorphosis, hampering pupation and causing abnormal wing development, reduced egg production, and reduced hatching rate. We believe that the misregulation of key hormone-related genes leads to abnormal pupa development in HDAC3 knockout insects. RNA-seq analysis identified 2788 differentially expressed genes (≥two-fold change; *p* ≤ 0.05) between siHDAC3- and siNC-treated larvae. Krüppel homolog 1 (Kr-h1), was differentially expressed in HDAC3 knockdown larvae. Pathway-enrichment analysis revealed the significant enrichment of genes involved in the Hippo, MAPK, and Wnt signaling pathways following HDAC3 knockdown. Histone H3K9 acetylation was increased in *H. armigera* after siHDAC3 treatment. In conclusion, HDAC3 knockdown dysregulated juvenile hormone (JH)-related and apoptosis-related genes in *H. armigera.* The results showed that the HDAC3 gene is a potential target for fighting *H. armigera*.

## 1. Introduction

Rapid advancements in biotechnological approaches have facilitated the application of genetic improvements to cope with pests and diseases. *H. armigera* Hübner (Lepidoptera, Noctuidae) is an omnivorous agricultural pest with a wide global distribution and more than 300 host species. It primarily harms crops such as cotton, corn, wheat, barley, tomato, green pepper, and melon, with massive losses to the agricultural economy [1]. Chemical pesticides, and the cultivation of transgenic plant lines expressing the *Bacillus thuringiensis* toxin, are two major strategies for controlling the pathogen. However, it has recently evolved resistance to chemical pesticides, and resistance genes associated with *B. thuringiensis* toxins have been detected in field populations, necessitating the development of novel pest control strategies [2].

In insects, double-stranded RNA (dsRNA) (first discovered in *Caenorhabditis elegans*) can be used to inhibit endogenous-gene expression [3], leading to phenotypic changes, which enables the study of gene function and the effects of gene deletion. DsRNA is widely applied in insect research, and dsRNA-based strategies are among the most common pest control methods [4]. Plant-mediated RNAi control of pests is considered as a novel and efficient strategy that is environmentally friendly and harmless to humans [5]. Small interfering RNA (siRNA), a type of dsRNA of 20–25 nucleotides, interferes with the transcription of genes with complementary nucleotide sequences, causing mRNA degradation and gene silencing [3,6]. It has advantages such as high specificity, efficiency, stability, heritability, and transferability. In *H. armigera*, however, dsRNA silencing of target genes is inefficient, primarily because Lepidoptera have relatively high levels of dsRNase activity, which can lead to dsRNA degradation more easily than siRNA [7,8]. In the case of *H. armigera*, therefore, it is more effective to select siRNA that specifically silences target genes. The level of gene silencing by RNAi varies with target genes and pests; consequently, selecting an appropriate target gene is challenging in pest control.

Precise regulation of gene expression is necessary for normal development and physiological function [9,10]. Histone acetyltransferases (HATs) and histone deacetylases (HDACs) maintain the dynamic balance of histone acetylation and deacetylation [11], participate in the regulation of chromatin compression, and control the assembly of the initiation complex on promoter DNA, affecting gene transcription [12,13]. HDACs belong to a class of epigenetic regulatory enzymes that reverse acetylation on the amino-terminal lysine residues of histones and restore their positive charge and intensify their tight entanglement with negatively charged DNA. Consequently, chromatin gets its dense and coiled structure, making it difficult for RNA polymerase to bind to gene promoters, thereby inhibiting gene transcription [14]. HDACs are divided into four families according to their structure, subcellular distribution, and enzymatic characteristics: Class I (HDAC1, HDAC2, HDAC3, and HDAC8), Class IIa (HDAC4, HDAC5, HDAC7, and HDAC9), Class IIb (HDAC6 and HDAC10), Class III (Sirt1-7), and Class IV (HDAC11) [15].

*HDAC3*, which differs from other class I HDACs, is the only one that functions by binding to the deacetylase-activating domain (DAD) of the NCoR (nuclear receptor co-repressor) and SMRT (silencing mediator of retinoic acid and thyroid hormone receptor) [12,16,17]. The NCoR/SMRT complex also contains TBL1X and TBL1XR1, which recognize histones and recruit the ubiquitination machinery and the 19S proteasome [18,19]. In cancer models, *HDAC3* are essential for the core regulation of transcription and cell proliferation [20]. *HDAC3* loss-of-function alleles are recessive lethal mutations during larval and pupal stages, accompanied by an increased expression of the pro-apoptotic gene HID, and play key roles in inhibiting apoptosis in *Drosophila* adult tissue [21]. In *Drosophila* S2 cells, RNAi-mediated *HDAC3* silencing results in cell-growth inhibition and abnormal expression of genes such as *sox14* and *eip74ef* [22]. In *Tribolium castaneum* larvae, RNAi-mediated *HDAC3* knockdown affects insect growth, development, and metamorphosis by altering histone acetylation levels, resulting in abnormal folding of adult wings [23], and the injection of dsHDAC3 causing significant larval and pupal mortality [24]. In *Aedes aegypti*, the knockout of four classes comprising 10 *HDAC*-homologous genes affected larval and pupal growth, and *HDAC3* knockdown increased apoptosis-related gene expression in the larval midgut, resulting in midgut damage, a severely abnormal phenotype, and 100% mortality [25].

Nonetheless, the function of *HDAC3* in *H. armigera* growth and development remains unclear. Therefore, in the present study, we analyzed its spatiotemporal expression in *H. armigera*. We further examined the effects of *HDAC3* loss-of-function by reducing *HDAC3* expression using of siRNA, and feeding larvae with RGFP966, a highly selective *HDAC3* inhibitor [26], on pupation, eclosion, egg laying, and hatching. RNA-seq analysis elucidated changes in gene expression associated with histone acetylation disturbances.

## 2. Results

### 2.1. Construction of HDAC3 Phylogenetic Trees

**Figure 1:**
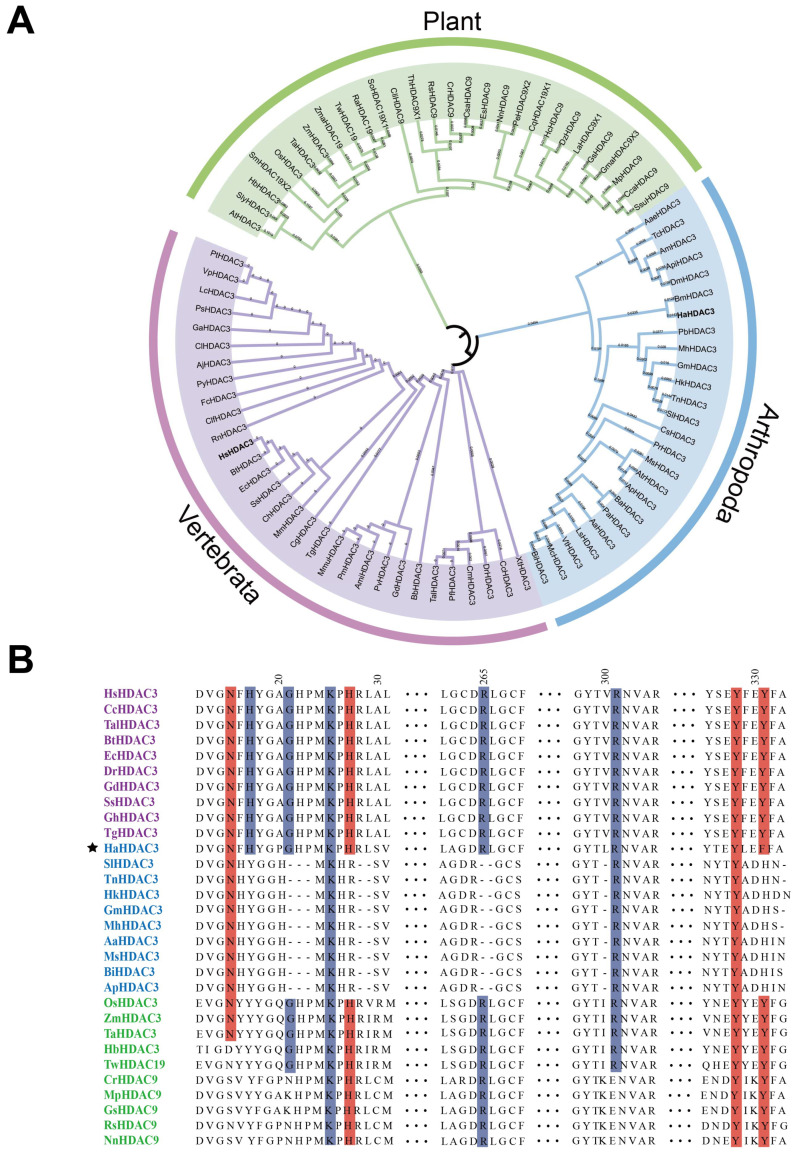
Phylogenetic analysis and sequence alignment of *HDAC3* in various species. (**A**) *HDAC3* protein sequences of various plants, arthropods, and vertebrates were aligned, and an NJ phylogenetic tree was constructed using MEGA-X. We used a bootstrap method with 1000 iterations to test the tree. (**B**) Ten sequences from each group (plants, arthropods, and vertebrates) were further analyzed. Residues that mediate interaction with Ins(1,4,5,6)P4 and SMRT-DAD are highlighted in blue and red, respectively. The star marks the *H. armigera*.

Based on human HDAC3 (GenBank: AAC98927.1) sequences, we searched the HDAC3 protein sequences from the NCBI database from 28 plants, 25 arthropods, and 31 (Table S1) vertebrate species (Figure 1A). Those vertebrates and arthropods were closely clustered in the same group while plant HDACs were closely clustered in one separate group. They mainly showed homology to the HDAC9 and HDAC19 and contained fewer HDAC3 sequences. These findings reveal that HDAC3 has evolved differently in plants, arthropods, and vertebrates.

To further interrogate the differences, we selected 10 sequences from each group (plants, arthropods, and vertebrates), and used DNAMAN 8.0 for sequence alignment. The alignment results were less consistent than expected. In particular, the HDAC3 sequence of *H. armigera* differed from those of other arthropods, being more similar to that of vertebrates (Figure 1B). The HDAC3 protein sequence of *H. armigera* has conserved sites that bind to Ins(1,4,5,6)P4 and SMRT-DAD [11], which are not found in other arthropods, but are widely present in vertebrates. Residues that mediate interaction with Ins(1,4,5,6)P4 and SMRT-DAD are highlighted in blue and red, respectively (Figure 1B). Consistent with our expectations, these findings indicate that the HDAC3 gene of *H. armigera* is evolutionarily clustered with those of arthropods, even though it has Ins(1,4,5,6)P4 and SMRT-DAD binding sites that are not found in those of other arthropods.

### 2.2. Analysis of HDAC3 Expression in Various Tissues and Developmental Stages

**Figure 2:**
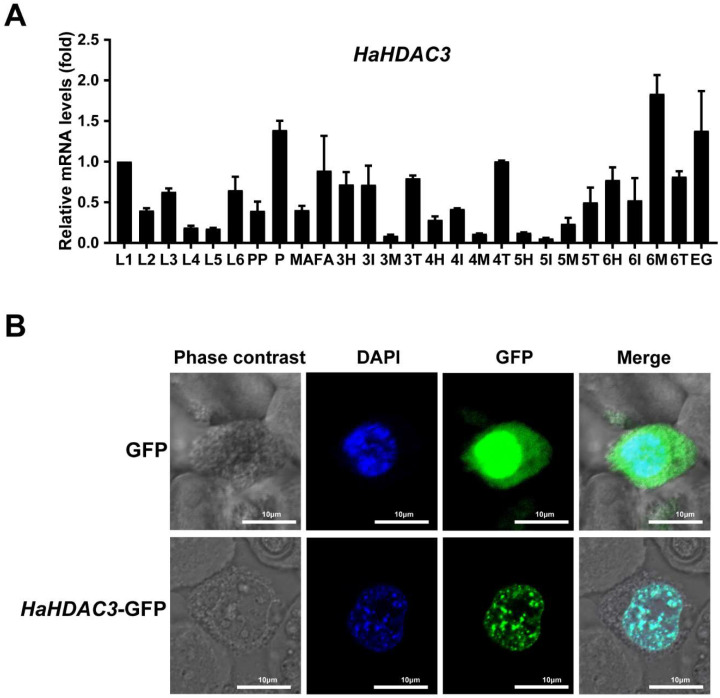
Spatial and temporal expression level and subcellular localization of *HDAC3* in *H. armigera*. (**A**) Larval stage (L1–L6), eggs (EG), prepupae (PP), pupae (P), female adults (FA), male adults (MA), head (H), integument (I), midgut (M), and malpighian tubules (T) (the numbers indicate the instar stages that used for sampling). Total RNA isolated from a pool of two larvae for each replication was converted to cDNA, and RT-qPCR was used to determine *HDAC3* mRNA relative expression. The mean ±SE (n = 4) is shown. (**B**) HaHDAC3 was transiently expressed in Sf9 cells to determine its subcellular localization. The green color shows the fluorescent signal of GFP expression. After staining with 4,6-diamidino-2-phenylindole (DAPI), the chromosome fluoresced blue, revealing the nuclear localization of HaHDAC3.

To examine *HDAC3* expression, we selected whole larvae at various instar stages (L1–L6), as well as eggs (EG), prepupae (PP), pupae (P), female adults (FA), and male adults (MA), along with head (H), integument (I), midgut (M), and Malpighian tubules (T) tissue from the instar stages 3–6 (Table S2). RT-qPCR revealed that *HDAC3* have the highest expression level in midgut of the 6th instar, pupae, and eggs (Figure 2A). As the 6th instar is larger and easier to inject, we selected it for siHaHDAC3 injection.

### 2.3. Subcellular HaHDAC3 Localization

To clarify *HDAC3* localization in Sf9 cells, we cloned the fusion protein GFP-*HDAC3* (KM983338.1) into a pFastBac baculovirus expression vector. Confocal laser scanning microscopy of the GFP-expressing control group cells revealed the fluorescence signal throughout the cells, whereas for the GFP-fused HaHDAC3-transfected cells, only the nucleus fluoresced (Figure 2B).

### 2.4. HDAC3 Is Required for Larval, Pupal, and Adult Survival

**Figure 3:**
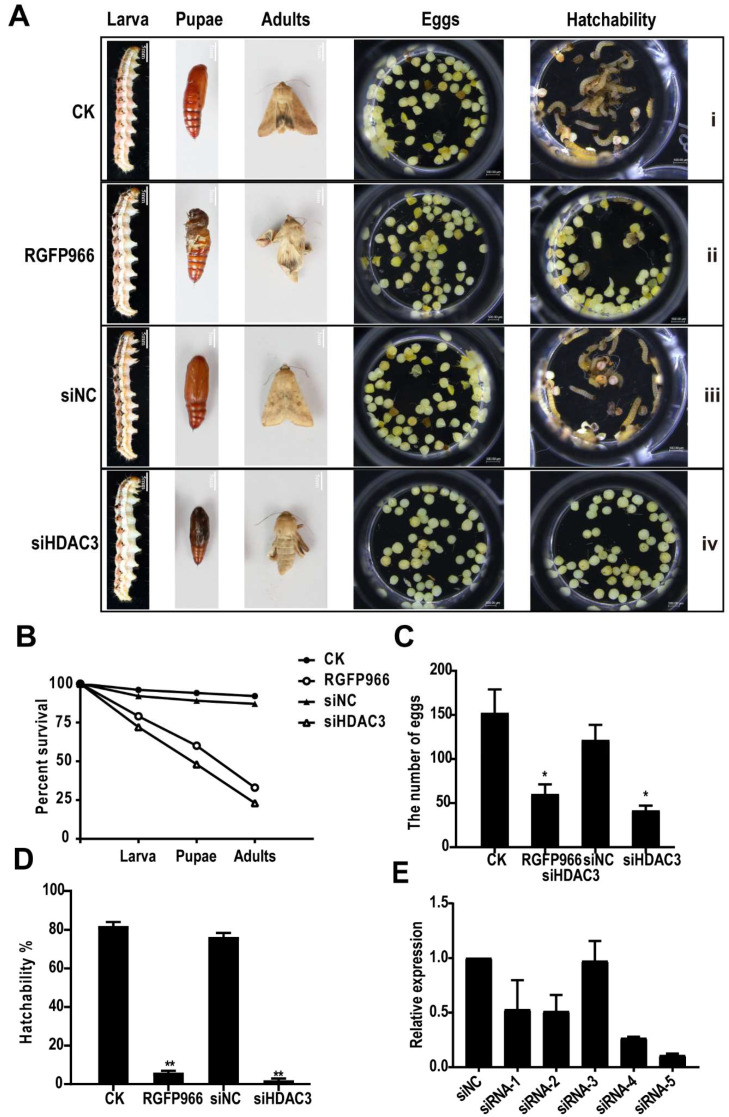
Phenotypes and mortality caused by *HDAC3* knockdown in *H. armigera*. (**A**) Phenotype at different developmental stages (pupal, adult, spawning, and hatching) after treatment. (i). Newly molted 5th instar larvae fed an artificial diet. (ii). Freshly molted 5th instar larvae fed an artificial diet containing 10 µM RGFP966, an *HDAC3*-inhibitor. (iii). Treatment of newly molted 6th instar larvae with siNC. (iv). Newly molted 6th instar larvae treated with siHDAC3. (**B**) Treatment lethality in larvae, pupae, and adults. Treatment effects on (**C**) the egg laying number, and (**D**) the hatching rate. (**E**) siRNA silencing efficiency (*t*-tests; * *p* < 0.05, ** *p* < 0.01).

Newly molted 5th instar larvae (100 *H. armigera* larvae in each group) were fed an artificial diet without RGFP966 inhibitor (RGFP966 is a class of highly selective inhibitors that interfere with HDAC3 function [26]) (control; Figure 3A(i)), or containing 10 µM of RGFP966 for 48 h [27] (Figure 3A(ii)). The control group grew, pupated, and emerged normally, whereas the treatment group showed developmental retardation at all stages, with a total mortality rate of 67% (comprising 21% larval mortality, 19% pupal mortality, and 27% adult mortality) (Figure 3B). Out of the 33% surviving individuals, the adults (one female and one male) were raised in a plastic box, and their egg-laying and hatching rates were counted. The females in the control group laid an average of 150 eggs, whereas the surviving inhibitor-group female laid an average of 60 eggs (Figure 3C). Fifty eggs were selected from each group; after 4 d, the hatching rates were 82% in the control and 6% in the inhibitor group (most of the unhatched eggs were nonviable), which was significantly lower than that in the control group (Figure 3D). Therefore, RGFP966 significantly inhibited normal growth, pupation, eclosion, egg laying, and hatching in *H. armigera*.

To obtain siRNA sequences with better silencing efficiency, we designed five siRNA sequences based on the *H. armigera HDAC3* gene sequence and compared their silencing efficiency. The five siRNA sequences (siRNA-1–5) were entrusted to Shanghai Sangon Biotech for synthesis (Table S3). At 24 h after injection, siRNA-4 and siRNA-5 exhibited better silencing efficiency, with that of siRNA-5 being outstanding (Figure 3E).

Therefore, we selected siRNA-5 for injection experiment. Each 6th instar larva of new molting is injected with 3 µ L siRNA-5 with a concentration of 1 µ g/µ L (Figure 3A(iv)). In the control group (Figure 3A(iii)), following the injection with a sequence targeting a *C. elegans* gene (siNC) (Table S3), most of the larvae pupated and emerged normally, whereas those injected with siHDAC3 exhibited abnormal growth and development at the larval, pupal, and adult stages, leading to death. In the adults, the wings were abnormally folded, making them unable to fly normally, with a total mortality rate of 77% (comprising 28% larval mortality, 24% pupal mortality, and 25% adult mortality). In contrast, the control group exhibited 8% larval mortality (Figure 3B). This includes larval mortality due to mechanical damage during the injection process. The egg-laying and the hatching rates were determined according to the same method used in the inhibitor experiment. On average, the control group females laid 121 eggs (Figure 3C) with a hatching rate of 76% (Figure 3D), while those in the siHDAC3 injection group laid 41 eggs (Figure 3D), and the hatching rate was significantly lower, at 2% (Figure 3D). Hence, both siHDAC3 injection and RGFP966 consumption severely inhibited growth, metamorphosis, and egg development severely.

### 2.5. HDAC3 Knockdown Affects Histone H3 Acetylation

**Figure 4:**
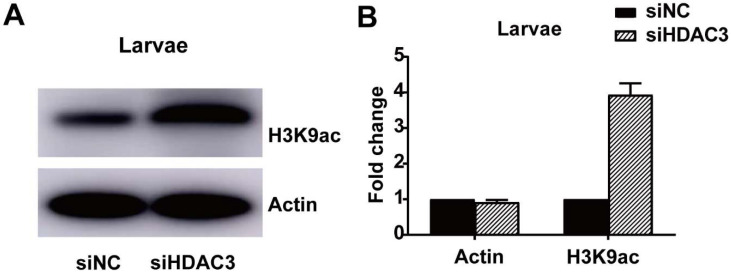
*HDAC3* knockdown affects H3 histone acetylation in *H. armigera*. (**A**) Total protein content was extracted from siHDAC3 and siNC-injected larvae, separated on SDS-PAGE gels, subjected to immunoblotting, and hybridized with an acetyl histone H3-recognizing antibody. β-Actin protein served as a control. HRP-linked IgG was used as secondary antibody. Histone H3K9 lysine acetylation was elevated in *HDAC3*-knockout larvae. (**B**) H3K9 levels were normalized against those of the loading control protein, β-actin. Band intensity was determined using ImageJ.

The total protein contents were isolated from the siHDAC3-treated final-instar larvae, and subjected to a Western blot assay, to determine *HDAC3* deacetylation targets. We evaluated the various lysine acetylation sites of histone H3 using Lys9, Lys14, Lys18, Lys27, and Lys56 specific antibodies. H3K9 acetylation was higher in siHDAC3-treated larvae than in the control (siNC-treated) larvae (Figure 4A,B).

### 2.6. Knockdown of HDAC3 Suppresses the Expression of Genes Involved in JH Action and Promotes the Expression of Genes to Apoptosis Pathway

**Figure 5:**
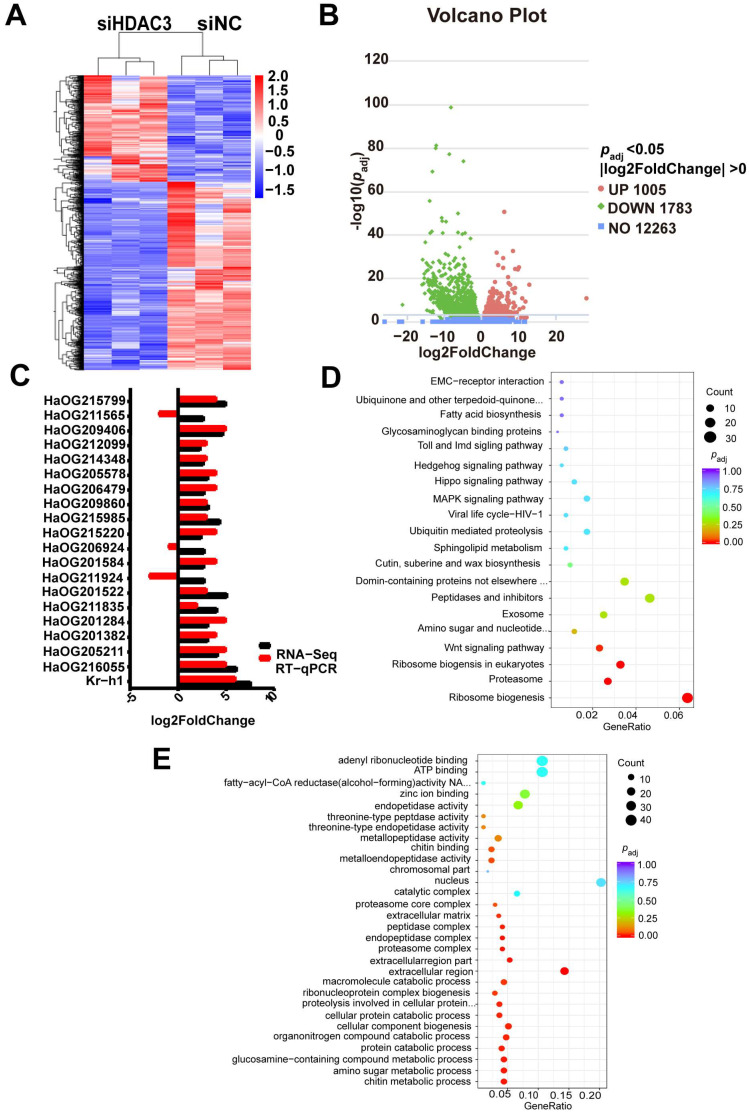
*HDAC3* knockdown affects the transcription of multiple pathway−related genes in *H. armigera*. (**A**) Heatmap of the RNA-seq normalized mean expression of 2788 differentially expressed genes (≥two-fold change; *p* < 0.05) between siNC−and siHDAC3−treated larvae. (**B**) Volcano plot of differentially expressed genes following *HDAC3* knockdown. *Y*-axis: −log10 P; *X*-axis: log2 fold change of mean normalized expression. (**C**) RT-qPCR verified gene expression pattern of 20 up-regulated DEGs of the RNA-seq data (Table S2). (**D**) The top 20 KEGG pathways enriched with DEGs. The *x*-axis represents GeneRatio. The specific pathway names were shown as following: ECM−receptor interaction, Ubiquinone and other terpenoid−quinone biosynthesis, Fatty acid biosynthesis, Glycosaminoglycan binding proteins, Toll and Imd signaling pathway, Hedgehog signaling pathway, Hippo signaling pathway, MAPK signaling pathway, Viral life cycle−HIV−1, Ubiquitin mediated proteolysis, Sphingolipid metabolism, Cutin, suberine and Wax biosynthesis, Domain-containing proteins not elsewhere classified, Peptidases and inhibitors, Exosome, Amino sugar and nucleotide sugar metabolism, Wnt signaling pathway, Ribosome biogenesis in eukaryotes, Proteasome, Ribosome biogenesis. (**E**) GO enrichment analysis of DEGs obtained from the comparison of siHDAC3 and siNA group of *H. armigera* larvae.

To identify the effects of HDAC3 knockdown on related genes and pathways, we sequenced RNA isolated from *H. armigera* with HDAC3 knockdown and control. The overall pattern of normalized mean expression values of differentially expressed genes (DEGs) is represented as a heatmap (Figure 5A). The differential gene expression analysis of RNA-seq data identified 1005 upregulated and 1783 downregulated genes in HDAC3 knockdown larvae compared to their expression levels in control larvae treated with siNC (Figure 5B; red and green dots in the volcano plots, respectively). These findings reveal significant differences in gene expression patterns between the treatment and control groups. Twenty genes (Table S2) with upregulated expression in RNA-seq results were selected for verification of RNA-seq data DEG predictions by RT-qPCR. Seventeen out of 20 genes tested showed an increase in their mRNA levels in HDAC3 knockdown larvae when compared to those in control siNC-treated larvae (Figure 5C). Notably, Kr-h1, a 20-hydroxyecdysone-dependent transcription factor with the ability to regulate larval metamorphosis development [28], was among the genes upregulated by the *HDAC3* knockdown.

Kyoto Encyclopedia of Genes and Genomes (KEGG) pathway enrichment analysis revealed that genes involved in the Hippo, MAPK, and Wnt signaling pathways were significantly enriched in *HDAC3*-knockdown samples (Figure 5D). Hippo signaling has emerged over the last decade as a major determinant of organ growth. Its role in growth control and tumorigenesis was first identified in *Drosophila* [29,30]. WEGO (Web Gene Ontology Annotation plot) showed enrichment of terms involved in the amino sugar metabolic process and cellular macromolecule catabolic process in the HDAC3 knockdown of insects (Figure 5E). Therefore, the *HDAC3* knockdown might induce apoptosis-related gene expression, resulting in larval death and abnormal development.

## 3. Discussion

The HDAC3 sequence alignment results reveal that HDAC3 in *H. armigera* differs from that of other arthropods, being more similar to that of vertebrates, retaining the residues of the interaction between HmHDAC3 and Ins(1,4,5,6)P4 and NCoR. As one of the many molecules recruited by the NCoR/SMRT complex, the catalytic function of HDAC3 needs to interact with the conservative domain of NCoR and SMRT proteins, which is called the deacetylase activation domain (deacetylase-activating domain, DAD) binding [17]. This domain is the key region in which NCoR/SMRT activates HDAC3 activity. There is a necessary Ins(1,4,5,6)P4 exist in HDAC3 and NCoR/SMRT complex, Ins(1,4,5,6)P4 acts as “intermolecular glue” to stabilize the interaction between HDAC3 and DAD by forming hydrogen bonds and salt bridges between two proteins. SMRT-DAD and Ins(1,4,5,6)P4 are necessary to activate the function of the HDAC3 enzyme [11]. HDAC3 is unstable without interaction with NCoR or SMRT [31]. The expression of both positively and negatively regulated genes by thyroid hormone (TH) in vivo is modulated by NCoR interaction with HDAC3 [32]. Previous studies demonstrated that the HDAC1/SIN3 multiprotein complex regulates the expression of Kr-h1 [23]. Further research on the NCoR-HDAC3 complex is needed to determine the gene regulatory mechanism of HDAC3.

Our studies reported that *H. armigera* HDAC3 is expressed during all developmental stages, and the highest mRNA levels were detected in 6th instar midgut (Figure 2A), As the 6th instar is larger and easier to inject, we selected it for siHDAC3 injection. The expression of HDAC3 in pupa and eggs is also relatively high, which indicates that HDAC3 plays an important role in the pupa and egg stages. The subcellular localization results showed that HDAC3 was localized in the nucleus. HDAC3 was a well-studied epigenetic factor, located on the mitotic spindle, which was necessary for the proliferation of neural stem cells, the G2/M phase of the mouse cell cycle that knocks out HDAC3 is blocked [33,34]. Our Western blot results showed that RNAi-mediated knockdown of HDAC3 results in an increase in acetylation of Histone H3K9 in *H. armigera* (Figure 4A). Similarly, in the Saccharomyces cerevisiae HDAC1 and RPD3 disruptions result in histone H3 hyperacetylation, especially at H3K9 and H4K12 [35]. Hyperacetylation of Lys-9 in histone H3 near the growth-differentiation factor 11 (gdf11) promoter was caused by being treated with HeLa cells with TSA or silencing of HDAC3 expression by small interfering RNA [36].

Knockout of HDAC3 gene from *A. aegypti* larvae increased the expression of apoptosis-related genes in the midgut and damaged the midgut, resulting in 100% mortality [25]. After knocking out the HDAC3 of *T. castaneum*, significant mortality was observed in the larval and pupa stages [24], some HDAC3 gene knockout larvae experience pupation, but the pupae show defects, especially the abnormal folding of wings [23]. In the *Gnatocerus cornutus*, RNAi-mediated HDAC3 knockdown caused a reduction in hind wing size [37]. One of the main results of this study is that HDAC3 is necessary for the normal development of larvae, pupae and adults of *H. armigera*. Treatment with HDAC3 siRNA and RGFP966, a HDAC3 inhibitor, increased mortality at each growth stage and affected metamorphosis, causing abnormal pupation, abnormal wing development, and reduced egg laying and hatching. Transcriptomic analysis revealed that HDAC3 knockdown dysregulated JH and 20-hydroxyecdysone (20E) genes. For instance, it significantly upregulates Kr-h1, Broad Complex (Br-C), and Ecdysone-induced protein 78C (E78). Kr-h1 has been known as a JH-early inducible gene responsible for the repression of metamorphosis, the expression of BR-C is induced by molting hormone 20E, and this induction is repressed by JH [28]. In *H. armigera*, the correct expression of Kr-h1 and Br-C is needed for larval-pupal-adult metamorphosis [38]. Knockdown of either Kr-h1 in *Pyrrhocoris* larvae causes precocious development of adult color patterns, wings, and genitalia [39]. Upon pupation, both BR-C and Kr-h1 are naturally downregulated by the absence of JH to allow adult development [40,41,42,43]. Based on these data, we believe that the misregulation of key hormone-related genes leads to abnormal pupa development in HDAC3 knockout insects. In general, histone acetylation and deacetylation in the promoter region are associated with transcriptional activation and transcriptional inhibition, respectively [44]. Interestingly, differential gene expression analysis of sequences of RNA isolated from siHDAC3 and siNC-treated larvae identified 1005 upregulated, and 1783 downregulated. These data indicate that HDAC3 is involved in the inhibition of gene expression in *H. armigera* larvae. Maintaining the balance between acetylation and deacetylation of histone and non-histone is essential for the healthy growth of cells. Loss of HDAC3 leads to the inhibition of cell growth and overexpression of genes involved in lipid metabolism, DNA replication, cell cycle regulation, and signal transduction [22]. Histone acetyltransferase and deacetylase control cell proliferation and differentiation [11].

Recently, functional genomics has facilitated the comprehensive study of physiological function in key pest genes, via the application of RNA interference (RNAi), whereby gene expression is downregulated using exogenous double-stranded RNA (dsRNA) [5]. Feeding with dsRNA could complement dsRNA injection for systemic gene silencing and reduce mortality and injection-induced damage [45]. Western corn rootworm larval mortality was high after feeding on transgenic maize plants expressing dsRNA against a western corn rootworm target gene [46]. In *H. armigera*, cytochrome *P450* gene silencing via transgenic plant-mediated RNAi impaired larval tolerance to a gossypol-containing diet. Further, the production of intact and long dsRNA strands by plants can inhibit gene expression in insects [47]. For *H. armigera*, although growth was inhibited, the method did not achieve pest control and high mortality. Based on recent research, dsRNA produced by *H. armigera* after feeding on transgenic plants is rapidly degraded in the intestinal fluid, whereas siRNA is relatively stable in the digestive system; in lepidopteran insects, siRNAs may be more effective than dsRNAs in triggering RNAi [7]. Therefore, the findings could optimize plant-mediated RNAi pest-control strategies. Furthermore, the results enhance our understanding of how RNAi participates in controlling lepidopteran pests, as well as a theoretical basis for constructing transgenic plants that silence the genes of lepidopteran pests.

In summary, *HDAC3* loss-of-function increased *H. armigera* mortality at each growth stage, and affected its metamorphosis and development, causing difficulty in pupation, abnormal wing development, and reduced egg laying and hatching. RNA-seq analysis of differential gene expression following *HDAC3* knockdown revealed dysregulation of genes related to 20-hydroxyecdysone and apoptosis. These findings indicate that *HDAC3* plays key roles in *H. armigera* growth, metamorphosis, and egg laying. *HDAC3* has been considered a good target and potential candidate for bio-based insecticide discovery. Therefore, the application of *HDAC3* for RNAi control in *H. armigera* opens the door to experiments with other members of the histone deacetylase family.

## 4. Materials and Methods

### 4.1. Phylogenetic Tree Construction and Sequence Alignment

A BLAST search (https://www.ncbi.nlm.nih.gov, accessed on 21 July 2021) using protein sequences homologous to human HDAC3 (GenBank: AAC98927.1) against plants, arthropod, and vertebrate sequences returned 28, 25, and 31 sequences (GenBank numbers are provided in Table S1), respectively, which were used to construct a phylogenetic tree. Amino acid sequences were aligned using MUSCLE in MEGA-X (Mega Limited, Auckland, New Zealand) [48]. A rootless maximum likelihood phylogenetic tree was constructed (1000 bootstrap iterations), and was visualized using iTOL (https://itol.embl.de, accessed on 15 November 2021). DNAMAN 8.0 (Lynnon Biosoft, San Ramon, CA, USA) was used to align 10 sequences selected from different clusters.

### 4.2. Insect Rearing and Feeding Experiments

Insect larvae and adults were cultured at 26 ± 1 °C, with a relative humidity of 60 ± 10% and a 16:8 h (light: dark) light cycle. The larval feed contained 33 g casein, 33 g D-glucose, 28 g wheat germ, 9 g Wesson salt mix, 5 g L-ascorbic acid, 4.7 g cellulose powder, 4.7 g sodium alginate, 1.8 g cholesterol, 1.5 g methyl paraber, 0.5 g sorbic acid, 1.1 g choline chloride, 2 g Vanderzant vitamin mixture, 0.5 g streptomycin, and 26 g Bacto Agar, and was added to 1 L water, stirred evenly, and boiled for 5 min. The feed was changed daily until pupation, and the adults were fed a 10% sucrose solution. Inhibitor-containing feed formulation: Add to feed with 1 μL/g RGFP966.

### 4.3. Analysis of HaHDAC3 Expression and Localization in Sf9 Cells

The GFP-*HDAC3* and GFP genes were cloned into a pFastBac baculovirus expression vector to identify the location of *HDAC3* in Sf9 cells. Construction vectors were generated using Phanta Max ultra-fidelity DNA polymerase (cat. no. P515-01; Vazyme, Nanjing, China) and verified via DNA sequencing. After fully mixing the constructed vector and transfection reagent, they were transfected into the Sf9 cell line, which was then cultured in a ESF 921 serum-free medium (Expression Systems, Davis, CA, USA) for 3–5 d. The slides containing the Sf9 cells were immersed in the culture plate with Phosphate-buffered Saline (PBS) thrice for 3 min each time. The slides were then fixed with 4% paraformaldehyde for 15 min, and were again immersed thrice for 3 min each time in PBS (containing 0.5% Triton X-100 permeabilized at room temperature [22–26 °C] for 20 min). The slides were again immersed in PBS three times for 3 min each time, blotted dry with absorbent paper, and the normal goat serum was added dropwise. The slides were then blocked at room temperature for 30 min. The blocking solution was absorbed using absorbent paper. Afterward, the appropriate quantity of the diluted primary antibody was dropped onto each slide, and the slides were placed in a wet box and incubated 12–14 h at 4 °C. The slides were then immersed in poly-butylene succinate-co-terephthalate (PBST) three times for 3 min each time, and were dried using absorbent paper. The diluted fluorescent secondary antibody was added dropwise, and the slides were incubated at 28–32 °C for 1 h in a wet box, washed thrice in PBST for 3 min each time, and then DAPI was added dropwise. The slides were incubated in the dark for 5 min, and were stained. Excess DAPI was washed off with PBST four times for 5 min each time. The slides were dried again using absorbent paper, and were sealed with an anti-fluorescence quencher. Images were obtained using a confocal laser scanning microscope (LSM 880, Carl Zeiss, Oberkochen, Germany).

### 4.4. Expression Pattern Analysis

RNA was extracted from tissue samples using a FastPure Cell/Tissue Total RNA Isolation Kit (Vazyme) according to the manufacturer’s instructions. One microgram of RNA was reverse transcribed into cDNA using the HiScript III 1st Strand cDNA Synthesis Kit (+Gdna wiper) (Vazyme), according to the manufacturer’s instructions. RT-qPCR analysis was performed using SYBR Green on a LightCycler 480 (Roche Diagnostics GmbH, Mannheim, Germany) (Table S2). The 20 μL reaction system comprised 2 μL (100 ng) cDNA, 0.4 μL forward primer (10.0 μmol/L), 0.4 μL reverse primer (10.0 μmol/L), 10 μL 2× ChamQ Universal SYBR qPCR Master Mix, and 7.2 μL nuclease-free water. The reaction program was as follows: 95 °C for 30 s; 40 cycles of 95 °C for 10 s, and 60 °C for 30 s. *HDAC3* expression was analyzed using the 2^−ΔΔCT^ method [49] using *RPS15* (GenBank: XM-021326200.1) as an internal control. Mean ± SE (n = 4) is shown. The significant differences were determined by *t*-tests (* *p* < 0.05, ** *p* < 0.01).

### 4.5. Small Interfering RNA (siRNA) Synthesis and Microinjection

Five HaHDAC3 (KM983338.1) siRNAs were designed using the GenScript tool (https://www.genscript.com/tools/sirna-target-finder accessed on 12 February 2022), and together with a *C. elegans* targeting siNC gene sequence (siRNA sequence information in Table S3), were entrusted to Sangon Biotech (Shanghai, China) for synthesis. Just-molted final-instar larvae were injected with siRNA using a microsyringe at 1 µg/µL (injection volume 3 µL) [23].

### 4.6. Protein Extraction

Total lysates were obtained via lysis in RIPA buffer with protease inhibitors for 30 min on ice. Cells were lysed in hypotonic lysis buffer containing 10 mM HEPES, 10 mM KCl, 1.5 mM MgCl, 0.5 mM dithiothreitol, and protease inhibitors. Afterward, 0.2 M H_2_SO_4_ was added, and the lysates were incubated at 4 °C for 30 min. After spinning, the proteins in the supernatant were precipitated using 33% trichloroacetic acid, washed with acetone, and resuspended in ddH_2_O. The protein extract was quantified using the BCA method [50].

### 4.7. Western Blot Analysis

The proteins were post-translationally modified by acetylation, which was detected using Acetylated-Lysine (Ac-K2-100) MultiMab^TM^ Rabbit mAb mix (Cell signaling #9814). Various lysine acetylation sites of histone H3 were detected by using histone H3 antibody sampler kits #9927 (Cell Signaling, Danvers, MA, USA) including Lys9, Lys14, Lys18, Lys27, and Lys56 specific antibodies. The band density was measured by Image-J software and normalized with the loaded control protein, β-Actin. Anti-rabbit IgG, HRP-linked antibody (Cell signaling #7074) was used for chemiluminescence detection.

### 4.8. Transcriptome Analysis

Total RNA was extracted from the siHDAC3 and siNC groups. The samples were subjected to gel electrophoresis, and transcriptome sequencing was performed at Beijing Nuohe Zhiyuan Technology Co. Ltd. (Beijing, China). The total RNA content of each sample is 3 micrograms, which is used as the input material for the preparation of RNA samples. Use the NEBNext^®^Ultra™RNA Library Prep Kit for Illumina ^®^to generate a sequencing library (NEB, Ipswich, MA, USA) and add the index code to the attribute sequence of each sample. TruSeq PE cluster toolkit v3-CBOT-HS (Illumina, San Diego, CA, USA) is a sample of cluster indicator coding used on the CBOT cluster generation system Raw Illumina reads have been deposited into NCBI’s (accession number: GSE215090) The gene ontology (GO) of DEG was condensed and analyzed by GO seq R software, and the gene length deviation was corrected. The GO term whose correction p is less than 0.05 is considered to be significantly rich in DEG. The statistical enrichment degree of DEG in the KEGG pathway was determined by using KEGG orthodontic marking system software.

## Data Availability

Not applicable.

## Supplementary Materials

The following supporting information can be downloaded at: https://www.mdpi.com/article/10.3390/ijms232314820/s1.
