# Supplementary material for: HDAC3 Knockdown Dysregulates Juvenile Hormone and Apoptosis-Related Genes in Helicoverpa armigera
Source: Int J Mol Sci. 2022 Nov 26;23(23):14820. doi: 10.3390/ijms232314820 (PMC9740019; doi:10.3390/ijms232314820)
Supplement: Supplementary file 1 [file ijms-23-14820-s001.zip › ijms-1994066-supplementary.pdf]

HDAC3-Knockdown Dysregulate Juvenile Hormone and Apoptosis-Related Genes in *Helicoverpa armigera*

Huimin Chang[1], Zhenlu Xu[1], Wenkang Li[2], Chenggu Cai[2], Wenjing Wang[1,2,3], Pengliang Ge[4], Xue Jia[1], Yingge Li[1], Tianze Ding[1], Wei Ma[5], Ali Mohammad Banaei-Moghaddam[6], Huijuan Mo[1,2*] and Maozhi Ren[1,2,3*]

Correspondence author: mohuijuan@caas.cn (H.M.); renmaozhi01@caas.cn (M.R.)

Table S1. The GenBank accession numbers of sequence used to construct the phylogenetic tree.

Abbreviations	GenBank	Species
AtHDAC3	AAG28475.1	Arabidopsis thaliana
SlyHDAC3	NP_001352081.1	Solanum lycopersicum
HbHDAC3	ASU54221.1	Hevea brasiliensis
SmHDAC19X2	XP_024526726.1	Selaginella moellendorffii
OsHDAC3	AAP47173.1	Oryza sativa Japonica Group
TaHDAC3	ABG43092.1	Triticum aestivum
ZmHDAC3	AAC50038.1	Zea mays
ZmaHDAC19	KMZ66840.1	Zostera marina
TwHDAC19	XP_038694164.1	Tripterygium wilfordii
RaHDAC19	XP_030552636.1	Rhodamnia argentea
SoHDAC19	XP_030450571.1	Syzygium oleosum
CliHDAC9	KAF3320763.1	Carex littledalei
ThHDAC9X1	XP_010541291.1	Tarenaya hassleriana
RsHDAC9	XP_018434438.1	Raphanus sativus
CrHDAC9	XP_006279377.1	Capsella rubella
CsaHDAC9	XP_010503116.1	Camelina sativa
EsHDAC9	XP_006419115.1	Eutrema salsugineum
NnHDAC9	XP_010243527.1	Nelumbo nucifera
PeHDAC9X2	XP_020571405.1	Phalaenopsis equestris
CqHDAC19X1	XP_021757988.1	Chenopodium quinoa
HcHDAC9	QBQ34150.1	Hibiscus cannabinus
DzHDAC9	XP_022755944.1	Durio zibethinus
LaHDAC9X1	XP_019453894.1	Lupinus angustifolius
GsHDAC9	XP_028190309.1	Glycine soja
GmaHDAC9X3	XP_003538135.1	Glycine max
MpHDAC9	RDY11714.1	Mucuna pruriens
CcaHDAC9	XP_020214800.1	Cajanus cajan
SsuHDAC9	TKY67096.1	Spatholobus suberectus
AaeHDAC3	XP_001655108.1	Aedes aegypti
TcHDAC3	XP_969419.1	Tribolium castaneum
AmHDAC3	XP_395811.2	Apis mellifera
ApiHDAC3	XP_001951271.1	Acyrthosiphon pisum
DmHDAC3	NP_651978.2	Drosophila melanogaster
BmHDAC3	XP_012552478.1	Bombyx mori
HaHDAC3	AJH76982.1	Helicoverpa armigera
PbHDAC3	XP_045518296.1	Pieris brassicae
MhHDAC3	XP_034827651.1	Maniola hyperantus
GmHDAC3	XP_026754881.1	Galleria mellonella
HkHDAC3	XP_026333196.1	Hyposmocoma kahamanoa
TnHDAC3	XP_026740888.1	Trichoplusia ni

SlHDAC3	XP_022831573.1	Spodoptera litura
CsHDAC3	RVE44174.1	Chilo suppressalis
PaHDAC3	XP_039752448.1	Pararge aegeria
MsHDAC3	KAG6455343.1	Manduca sexta
AtrHDAC3	XP_013193311.1	Amyelois transitella
ApHDAC3	CAB3219866.1	Arctia plantaginis
BaHDAC3	XP_023947594.1	Bicyclus anynana
PaHDAC3	XP_039752448.1	Pararge aegeria
AaHDAC3	XP_041972205.1	Aricia agestis
LsHDAC3	VVD00622.1	Leptidea sinapis
VtHDAC3	XP_026489054.1	Vanessa tameamea
McHDAC3	XP_045454150.1	Melitaea cinxia
BiHDAC3	CAH0713927.1	Brenthis ino
XtHDAC3	NP_001016883.1	Xenopus tropicalis
CcHDAC3	XP_041049995.1	Carcharodon carcharias
DrHDAC3	NP_957284.1	Danio rerio
CmHDAC3	XP_036425068.1	Colossoma macropomum
PfHDAC3	XP_039668920.1	Perca fluviatilis
TalHDAC3	XP_044218859.1	Thunnus albacares
BbHDAC3	XP_040297402.1	Bufo bufo
GdHDAC3	NP_990078.2	Gallus gallus domesticus
PvHDAC3	XP_020654852.1	Pogona vitticeps
AmiHDAC3	XP_006265553.1	Alligator mississippiensis
PmHDAC3	XP_015684433.1	Protobothrops mucrosquamatus
MmuHDAC3	XP_044883899.1	Mauremys mutica
TgHDAC3	XP_025244269.1	Theropithecus gelada
CgHDAC3	XP_003508244.1	Cricetulus griseus
MmHDAC3	XP_028705021.1	Macaca mulatta
ChHDAC3	XP_005683119.1	Capra hircus
SsHDAC3	NP_001230756.1	Sus scrofa
EcHDAC3	XP_001504028.1	Equus caballus
BtHDAC3	NP_001193172.1	Bos taurus
HsHDAC3	AAC98927.1	Homo sapiens
RnHDAC3	NP_445900.2	Rattus norvegicus
ClfHDAC3	XP_038514649.1	Canis lupus familiaris
FcHDAC3	XP_011280921.3	Felis catus
PyHDAC3	XP_040310374.1	Puma yagouaroundi
AjHDAC3	XP_014918986.1	Acinonyx jubatus
ClHDAC3	XP_005380780.1	Chinchilla lanigera
GaHDAC3	XP_044518501.1	Gracilinanus agilis
PsHDAC3	XP_032484400.1	Phocoena sinus
LcHDAC3	XP_030176237.1	Lynx canadensis
VpHDAC3	XP_006204584.1	Vicugna pacos
Pt HDAC3	XP_007077979.1	Panthera tigris

Table S2. List of primers used in this work.

Gene	Primer
qHaHDAC3-57-F	CCGTTGAAAGAAGGGATTGA
qHaHDAC3-57-R	GCGTTGATAGCGAGAAGCA
RPS15-F	CTGAGGTCGATGAAACTCTC
RPS15-R	CTCCATGAGTTGCTCATTG
B5X24_HaOG203563-F	CGTTGTGAATGGGAATGTTATG
B5X24_HaOG203563-R	CAGCGAGGGAGAGGGTGA
B5X24_HaOG216055-F	TCAGACAATCCAGCCACTACCC
B5X24_HaOG216055-R	GTCCAAATACGAGCATAACCAT
B5X24_HaOG205211-F	GTTTGGCTAAGGATGACACGC
B5X24_HaOG205211-R	CCGAGAAAGGTATGAGAACAGGT
B5X24_HaOG201382-F	CGAAGTACCCAGCAACAAAGA
B5X24_HaOG201382-R	TGGTCCAAATACGAGCATAAC
B5X24_HaOG201284-F	GACCTTCGGTGGAGCAGATAA
B5X24_HaOG201284-R	TACTTTTGTTGTTTCCTCGTCTAAT
B5X24_HaOG211835-F	GGATTCCAGGAGCAACCAC
B5X24_HaOG211835-R	GGGCACTTGAAACCCGTAG
B5X24_HaOG201522-F	TGTGACACCAGCAAATGAATCT
B5X24_HaOG201522-R	TCTAAATGCGGCAGGTAAGC
B5X24_HaOG211924-F	CGTTGTGGGCTATGACCTGT
B5X24_HaOG211924-R	AATGTGGGACTGTCGTGCTG
B5X24_HaOG201584-F	GTATGTGACCGAGGAACGAAT
B5X24_HaOG201584-R	CGATGATGTAGAACTGAGCGA
B5X24_HaOG206924-F	GAAGACCAAACACGAGGCT
B5X24_HaOG206924-R	CAGAGGCAGTCCAGAACGA
B5X24_HaOG215220-F	CTGGAAACAACGGGAATGG
B5X24_HaOG215220-R	CGGAGGCTGTATGAAGGGC
B5X24_HaOG215985-F	ATGGTGGCTATGGACCTTACA
B5X24_HaOG215985-R	GGTCCGATGTACTCGTAGAAGC
B5X24_HaOG209860-F	ATGCGAGATTGGATGATAAGAAAG
B5X24_HaOG209860-R	TGAAGTAGTGTTGGCAAGTAGACG
B5X24_HaOG206479-F	ACAGGAGATGCTCGCAATAGG
B5X24_HaOG206479-R	TGGGTCCATCAACGAATCTG
B5X24_HaOG205578-F	TATCACATAGGTCCGCAGCAT
B5X24_HaOG205578-R	TTCACCCGCAATCCAAGTC
B5X24_HaOG214348-F	CGCCGTGCCACCATACTT
B5X24_HaOG214348-R	CACTGCCGCCGCTGA
B5X24_HaOG212099-F	TTCCCACCAGACTACCCATACT
B5X24_HaOG212099-R	TGAGATCACGGATAGCAGCAC
B5X24_HaOG209406-F	AAGTTATCTTTGTGGCTTATTGTCG
B5X24_HaOG209406-R	GCTGAGCAATGGACGGACTAA
B5X24_HaOG211565-F	ACGCCCGCAGTAGTCTCCAA
B5X24_HaOG211565-R	GGCTCTGTTCCGAAGTATCCTGTT
B5X24_HaOG215799-F	AAAGCATAAGAAAGCCAACAAG
B5X24_HaOG215799-R	TGGAGCAGGTCGGGTAGAT

Table S3. siRNA sequence information.

Name	Sequence
siRNA-1：sense:	5’-AAGGAACCACCAGUUGAGAAC-3’
siRNA-1：Anti-sense:	5’-GUUCUCAACUGGUGGUUCCUU-3’
siRNA-2：sense:	5’-AAGAACGAAUUCUAUGAAGAC-3’
siRNA-2：Anti-sense:	5’-GUCUUCAUAGAAUUCGUUCUU-3’
siRNA-3：sense:	5’-AACUGGUCCGGAGGUCUACAU-3’
siRNA-3：Anti-sense:	5’-AUGUAGACCUCCGGACCAGUU-3’
siRNA-4：sense:	5’-AACAUGUGUACGACAACCUCA-3’
siRNA-4：Anti-sense:	5’-UGAGGUUGUCGUACACAUGUU-3’
siRNA-5：sense:	5’-AAACCAUAUAGAGCCAGUGCU-3’
siRNA-5：Anti-sense:	5’-AGCACUGGCUCUAUAUGGUUU-3’
siNC：sense:	5’-UUCUCCGAACGUGUCACGUTT-3’
siNC：Anti-sense:	5’-ACGUGACACGUUCGGAGAATT-3’
